# Approach to Endocrine Hypertension: A Case-Based Discussion

**DOI:** 10.1007/s11906-025-01323-w

**Published:** 2025-01-16

**Authors:** Sanja Borozan, A. B. M. Kamrul-Hasan, Sahana Shetty, Joseph M. Pappachan

**Affiliations:** 1 Department of Endocrinology, Clinical Centre of Montenegro, Podgorica, Montenegro; 2 Faculty of Medicine, University of Montenegro, Podgorica, 81000 Montenegro; 3 Mymensingh Medical College, Mymensingh, Bangladesh; 4 Department of Endocrinology, Kasturba Medical College, Manipal Academy of Higher Education, Manipal, 576104 India; 5 Faculty of Science, Manchester Metropolitan University, Manchester, M15 6BH UK

**Keywords:** Endocrine hypertension, Resistant hypertension, Primary aldosteronism, Aldosterone renin ratio, Pheochromocytoma, Cushing syndrome, Acromegaly

## Abstract

**Purpose of Review:**

Hypertension remains a major chronic disease morbidity across the world, even in the twenty-first century, affecting ≈40% of the global population, adversely impacting the healthcare budgets in managing the high incidence of cardiovascular disease (CVD) complications and mortality because of elevated blood pressure (BP). However, evaluation and management of endocrine hypertension are not optimal in clinical practice. With three unique clinical case scenarios, we update the evidence base for diagnostic evaluation and management of endocrine hypertension in this review to inform appropriate day-to-day clinical practice decisions.

**Recent Findings:**

Although most individuals with high BP suffer from essential hypertension (≈85%), some patients may have a clear underlying etiology (termed secondary hypertension), and a significant proportion of these patients have endocrine hypertension (≈10%) consequent to hormone excess from dysfunction of one or more endocrine glands. Even if a relatively common disease in the general population, the correct diagnosis and appropriate treatment of endocrine hypertension is often delayed because of poor awareness among clinicians, including primary care providers and physicians in the secondary care settings.

**Summary:**

An accurate and timely diagnosis of endocrine hypertension is crucial to potentially cure or at least properly manage these patients because the consequences of delays in diagnosis can be catastrophic, with markedly higher end-organ complications such as CVD, chronic kidney disease, and even premature mortality among sufferers.

## Introduction

Hypertension is one of the most common chronic disease morbidities, affecting ≈40% of the world population, tremendously increasing the cardiovascular disease (CVD) burden across the globe [1]. Although most patients with hypertension have essential hypertension, ≈15% of cases have secondary hypertension with a clear underlying cause, potentially curable by early diagnostic workup and institution of appropriate management [2]. However, many of these cases are either ignored by the patient or underdiagnosed by their physicians, which can result in premature CVD and death from a complication of hypertension.

Of the cases with secondary hypertension (SH), a significant proportion of patients have endocrine hypertension from various hormonal abnormalities. Of these causes, primary aldosteronism (PA) forms the vast majority of cases, although most practitioners, including physicians and endocrinologists, often miss the diagnosis. Most of the other causes of endocrine hypertension are rather uncommon. Recent evidence suggests that about 10% of hypertensives in the general population and ≈20% of patients attending hypertension clinics in secondary care settings have PA as the cause of their disease [3]. Timely diagnosis of an endocrine pathology in patients with hypertension is crucial to institute an appropriate management strategy on time that can be very rewarding as a good proportion of these patients gets a complete cure or good control of the disease, which markedly reduces the risk of catastrophic hypertensive complications. With three unique case scenarios, we elaborate on the pragmatic approach to disease evaluation with a brief discussion on management for patients with endocrine hypertension in this evidence-based review.

## Case 1

A 32-year-old female was referred to the endocrine clinic for evaluation of possible secondary hypertension. Hypertension was initially diagnosed at the age of 29 years during her 3rd pregnancy. Her 4th pregnancy was complicated by preeclampsia, for which she underwent a cesarean section at 37th weeks of gestation. Her blood pressure (BP) control was inadequate (170/110 mm Hg) while on daily treatment with amlodipine 10 mg and ramipril 10 mg. Her father had sudden cardiac death at 41 years with a history of hypertension. Her serum potassium was 3.4 mmol/L, sodium 139 mmol/L, and creatinine 46 micromol/L. Her hormonal evaluation showed plasma aldosterone concentration (PAC) of 514 pmol/L (normal: 0–1200), with a suppressed renin < 1.8 mIU/L (5.3- 99 mIU/L) level, and a very high aldosterone renin ratio (ARR; the ARR was incalculable due to unmeasurably low renin; normal value: < 30 pmol/L per mIU/L) suggesting probable primary aldosteronism (PA). Although PAC was within the normal laboratory range, suppressed renin level with a very high ARR raised the high likelihood of PA. Her random serum cortisol and plasma free metanephrines were within normal range.

After switching over antihypertensive drugs to hydralazine 50 mg TDS, Doxazosin 12 mg daily, and verapamil 240 mg daily over 3 months period (to avoid drug interference with ARR assays), a sitting saline suppression test (infusion of 2 L of 0.9% saline in 4 h with baseline blood samples prior and at the end of 4 h) was performed for biochemical confirmation of PA. The baseline values for aldosterone, renin and ARR were 373 pmol/L, 10.1 mIU/L and 36.9 pmol/L per mIU/L, respectively, while at the end of the test 461 pmol/L, renin 7.2 mIU/L and 64.02 pmol/L per mIU/L, respectively, indicating autonomous aldosterone excess with the confirmation of PA. An abdominal computed tomography (CT) scan showed a 1 cm left adrenal nodule measuring –10 Hounsfield units (HU), consistent with a lipid-rich adenoma. The patient underwent laparoscopic left adrenalectomy, which led to the normalization of PAC and hypertension, currently off all antihypertensives.

## Case 2

A 34-year-old male with previously diagnosed hypertension (BP: 180/120 mm Hg on routine health check-up) was admitted to the hospital for acute onset of shortness of breath while climbing stairs followed by dizziness and a pre-syncopal episode. His mother had a total thyroidectomy in the past (details not known). At admission, he was tachycardic (pulse 115/min), hypertensive (BP: 217/145 mm Hg), and tachypneic (respiratory rate: 20/min, SpO2 100% on room air). The laboratory findings were: leucocytosis (white cell count 15.2 × 10^9^/L) and raised random glucose (9.9 mmol/L), with normal creatinine, C-reactive protein, calcium, and phosphate levels. Blood gas analysis showed alkalosis (pH 7.62), with PaCO2 – 3.10 kPa, PaO2 – 8.61 kPa, bicarbonate 24 mmol/L and lactate of 2.4 mmol/L.

**Fig. 1:**
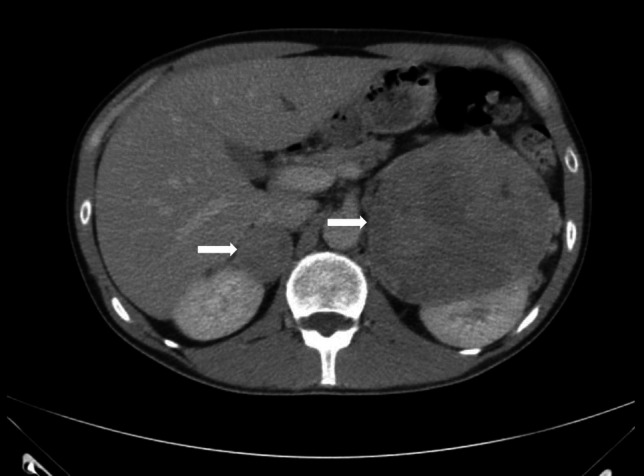
CT scan showing bilateral adrenal masses

**Fig. 2:**
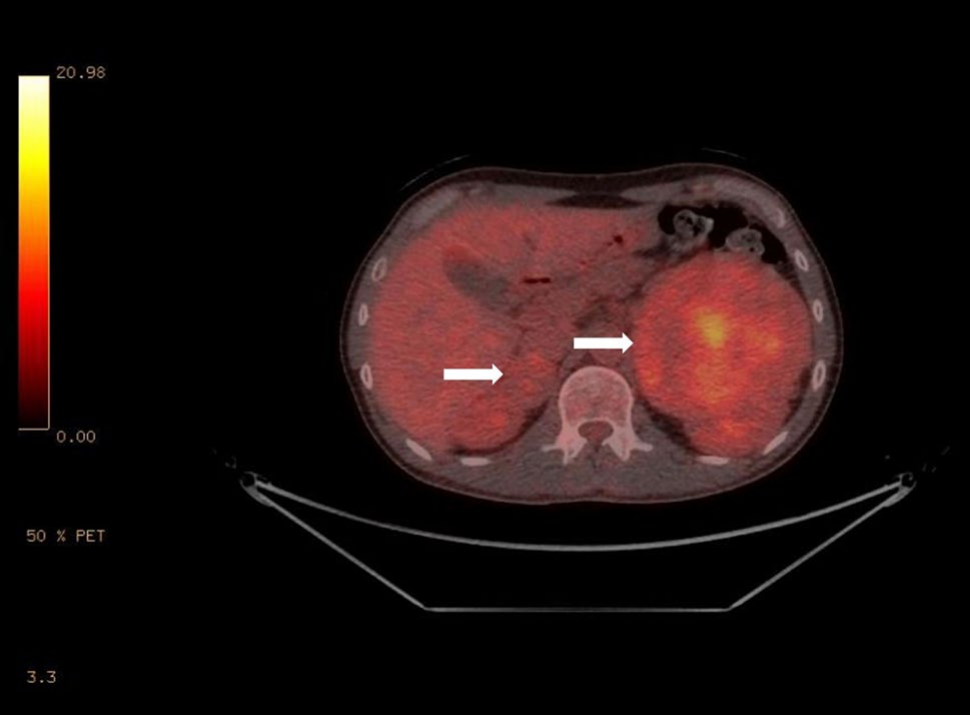
FDG- PET scan showing bilateral adrenal masses with tracer uptake

Given the clinical presentation, a CT pulmonary angiography scan was ordered to exclude pulmonary embolism that revealed bilateral inhomogeneous masses in the suprarenal region (Fig. 1*** arrows).*** The larger left mass was predominately solid, with cystic elements measuring 11.1 × 8 cm (average pre-contrast radiodensity: 48 Hounsfield Units [HU]), causing displacement and compression of the upper pole of the left kidney, and the right adrenal mass was 3.5 × 4 cm (36 HU). Urinary metanephrines using high-performance liquid chromatography (HPLC) showed very highly elevated urine metadrenaline of 390 umol/24 h (Normal: < 1.3 umol/24 h) and normetadrenaline of 255 umol/24 h (Normal: < 3.7 umol/24 h) confirming the biochemical diagnosis of pheochromocytoma. According to the size of both adrenal masses, metaiodobenzylguanidine (MIBG) scintigraphy and positron emission tomography (PET) were performed to rule out metastatic/multifocal disease. The tracer uptake in PET (Fig. 2*** arrows***) was consistent with bilateral pheochromocytoma and without any metastatic lesions detected. The patient’s hypertension was controlled with phenoxybenzamine with the subsequent addition of bisoprolol, and he underwent bilateral subtotal adrenalectomy. The adrenal insufficiency after surgery was managed by hydrocortisone and fludrocortisone replacement. Due to his bilateral adrenal masses and the history of thyroidectomy in his mother, genetic testing for multiple endocrine neoplasia type 2 (MEN 2) was performed. The analysis detected a heterozygous mutation in the *RET* protooncogene, confirming the diagnosis of MEN 2. Four weeks after surgery, the urine metanephrines were within the reference range, and his BP was normal without any antihypertensive drugs. Because of his hereditary syndrome, the patient is under regular lifelong follow-up.

## Case 3

**Fig. 3:**
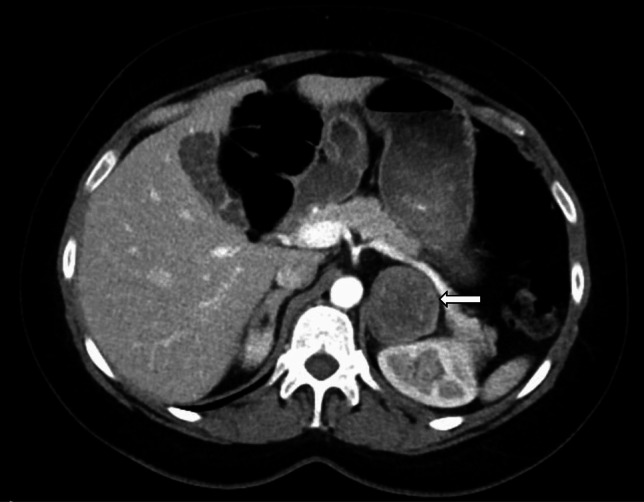
CT scan showing a left adrenal mass

A 31-year-old female with a history of Kawasaki disease and metabolic dysfunction-associated fatty liver disease (MAFLD) presented in the Emergency department in the 39th week of her first pregnancy with sudden onset headache and dizziness. She was hypertensive (144/108 mm Hg) and tachycardic (heart rate [HR] 107/min). Laboratory findings were unremarkable except for elevated alkaline phosphatase of 185 IU/L (normal: 30–130 IU/L), and alanine transaminase (ALT) 76 U/l (4—36 U/L). The labor was induced, and uncomplicated vaginal delivery occurred the same day. Hypertension was initially treated with labetalol and then with a combination of ramipril, amlodipine, and nebivolol. Eleven months later, she was readmitted with abdominal pain radiating to her back and multiple episodes of vomiting. BP was 162/104 mm Hg, HR 106/min, while abdominal ultrasound revealed multiple gall stones and an incidental left suprarenal mass. An abdominal CT scan confirmed a 4.5 × 4.2 cm mass in the left suprarenal gland (Fig. 3*** arrow***), and the right adrenal gland also appeared mildly enlarged.

An evaluation for adrenal incidentaloma suggested Cushing’s syndrome (CS) with unsuppressed cortisol (2649 nmol/L; normal < 50) in the overnight dexamethasone suppression test (ODST). Baseline adrenocorticotropic hormone (ACTH) was high: 472 pg/mL (normal < 46 pg/ml), with a 24-h urine cortisol > 6200 nmol (0–162 nmol/24 h). Other abnormal hormone assays were: plasma metadrenaline – 1.55 nmol/L (< 0.51 nmol/L), noradrenaline – 16.90 nmol/L (< 1.18 nmol/L), and elevated levels of testosterone, androstenedione and dehydroepiandrosterone sulfate (DHEAS) levels (4.1 nmol/L, 25.2 nmol/l, and 23.8 umol/l respectively), with a plasma free androgen index (FAI) of 24.7. The raised ATCH levels with hypercortisolemia would normally suggest ACTH-dependent CS from an ACTH-oma arising from the pituitary gland or ectopic ACTH production from various extra-pituitary sites (rarely from a pheochromocytoma). However, the MRI of her pituitary gland and CT scan of the chest were normal raising the possibility of an adrenal source of ACTH and catecholamine excess from the left adrenal tumor. With a presumptive diagnosis of ectopic ACTH production from a pheochromocytoma, patient was recommended left adrenalectomy.

After prompt preoperative treatment with metyrapone and phenoxybenzamine, for optimal control of cortisol and catecholamine excess with good control of hypertension, the patient underwent unilateral adrenalectomy. She was discharged on hydrocortisone 10 mg in the morning with 5 mg at lunch and 5 mg in the evening for transient adrenal insufficiency that usually follows surgical cure of adrenal Cushing’s syndrome. The postoperative plasma metanephrines were normalized when checked 2 weeks later with the complete resolution of her hypertension (BP: 115–120/75–85 mm Hg without antihypertensives). The final diagnosis in this unusual case was pheochromocytoma co-secreting ACTH with resultant CS.

## Discussion

The three cases presented above highlight the challenging process of establishing the diagnosis and identifying the unique underlying pathology in patients with endocrine hypertension. Further, case management exemplifies the gratifying results of a complete cure of the disease process by appropriate treatment, resulting in desired clinical outcomes. It is crucial for clinicians to have a basic understanding of these disorders for appropriate diagnostic evaluation and to plan for the most desirable treatment on time to avoid catastrophic complications from the underlying disease.

### When to Suspect Endocrine Hypertension?

Despite increasing awareness among clinicians, endocrine disorders remain a significant and often neglected cause of SH. The most common causes of endocrine SH are associated with adrenal gland pathology, but more than 15 different disease entities that affect the endocrine system can cause hypertension [4]. SH phenotypes pose a high cardiovascular risk, and the potential curability is the most significant advantage of establishing the underlying cause of hypertension [5, 6]. On the other hand, delayed diagnosis and treatment of secondary hypertension could have deleterious consequences on vital organs. For that reason, clinicians should know when to suspect and actively search for the possible underlying cause of hypertension that will further allow the initiation of adequate therapy. An appropriate case selection is crucial for rationalizing biochemical and hormonal testing and further imaging procedures to avoid unnecessary over-investigations and resource misuse.

Endocrine hypertension may be easily recognized in patients with unique features of hormonal disturbance such as acromegaly, cortisol excess, or pronounced thyroid disorders [7]. However, the symptoms and signs can often be subtle or nonspecific, which can often result in delays in diagnosis. According to the European Societies of Cardiology and Hypertension (ESC/ESH) guidelines, the high-risk hypertensive group of patients in whom the screening for SH is recommended include younger age (below 40 years), acute worsening of BP regulation in previously stable hypertension, severe disease (grade 3 hypertension; BP ≥ 180/120 mm Hg), resistant hypertension, or the presence of extensive hypertension-mediated organ damage [2, 8]. Resistant hypertension is defined as failure to achieve BP below 140/90 mm Hg despite simultaneous use of at least three antihypertensive drugs from different classes, including a diuretic in maximum doses [9]. SH is much more frequent in individuals with resistant hypertension, with a reported prevalence of 31% [10], compared to 15% in the general hypertensive population [2].

The distinctive clinical constellations and typical paroxysms can point to the right diagnosis, as in pheochromocytoma. Furthermore, some laboratory findings (like spontaneous hypokalemia in the absence of any apparent cause) or incidental tumor mass in the suprarenal gland may guide clinicians to investigate the presence of endocrine SH [2].

### Primary Aldosteronism

The diagnostic and therapeutic landscape of primary aldosteronism (PA) has changed tremendously since the first reports of the disease, particularly in the last two decades. Initially, PA was considered to be a rare cause of hypertension, accompanied by hypokalemia, periodic muscle weakness, and metabolic alkalosis [11]. From that point, it took a long way to the modern approach, which perceives PA as a syndrome, a spectrum of disorders characterized by inappropriately high, non-suppressible, and renin-independent aldosterone production, with suppressed baseline renin secretion, or the incapability to stimulate renin secretion [12].

Recently, the 2022 WHO Classification of Adrenal Cortical Tumors proposed a classification of morphological substrates for PA: aldosterone-producing adrenal cortical adenoma (APA), diffuse adrenal cortical hyperplasia (APDH), adrenal cortical nodule (APN), adrenal cortical micronodule (APM), multiple APN or APM (MAPN/MAPM) and adrenal cortical carcinoma (APACC) [13].

Most cases of PA are sporadic, with only 1 – 5% of individuals having the genetic basis [14]. The wide variation in the reported prevalence of familial hyperaldosteronism (FH) is due to the selection bias in genetic testing of patients from different series reported from various regions worldwide. Four forms of FH are recognized, with type I (glucocorticoid remediable aldosteronism) due to a hybrid *CYP11B1/CYP11B2* gene being the most common [15, 16]. Table 1 provides a brief description of the four forms of FH.

**Table 1:** Classification of the types of Familial hyperaldosteronism (FH) with common clinical features, genetic defects, type of inheritance and management options^2,14^. GRA – glucocorticoid remediable aldosteronism, AD – autosomal dominant, PA – primary aldosteronism, MRA – mineralocorticoid receptor antagonist, CCB – calcium channel blocker

FH type	Defective gene	Inheritance pattern	Common clinical picture	Treatment options
FH-I or GRA	*CYP11B1/CYP11B2*	AD	Early-onset of severe PA usually before the age of 20 years	Glucocorticoids and/or MRA treatment
FH-II	*CLCN2*	AD	Early-onset of PA and variable presentation of the disease severity depending on the penetrance of the mutation	Hypertension managed by MRA or other antihypertensive agents
FH-III	*KCNJ5*	AD	Early onset of severe PA with bilateral massive adrenal hyperplasia	MRA for hypertension management. Severe cases may require bilateral adrenalectomy for disease control
FH-IV	*CACNA1H*	AD	Early onset of PA with developmental delay in affected children	MRA with/without CCB for managing hypertension

Since aldosterone is the main regulator of water and electrolyte balance, the connection between abnormal hypersecretion and hypertension is clear. Besides that, the harmful effects of PA extend beyond hypertension and its direct consequences on organs since aldosterone promotes fibrosis, inflammation, and oxidative stress in specific target tissues, with subsequent renal and CV injury [17, 18].

Despite its clinicopathological effects and the associated complications, PA remains largely underdiagnosed, with less than 2% of people among the at-risk populations even tested for the disease [19]. However, several recent publications on PA have helped to raise global awareness. [2, 12, 15, 20] With improved disease recognition coupled with more accurate diagnostic procedures, the care of patients with the disease could improve further in the coming years.

A systematic review and meta-regression analysis by Kayser et al. showed a huge heterogeneity in the prevalence figures among hypertensive patients, depending on the study population: from 3.2% to 12.7% in primary care and from 1% to 29.8% in referral centers [21]. Almost every patient with PA has hypertension [16], while PA is the most common cause of SH [15]. On the other hand, hypokalemia, traditionally used as a hallmark of the disease, can be found only in 9–37% of all cases of PA, mostly in patients with aldosterone-producing adenoma, and it relates to worse CV outcomes [22]. Current understanding of PA expanded a population group in which an active search for the disease is advised. Thus, the high-risk group patients for PA include those with resistant or severe hypertension or hypertension with hypokalemia, adrenal mass, atrial fibrillation, sleep apnea, or a family history of the disease [12].

According to the guidelines, the gold standard for case detection as the first step includes the determination of plasma aldosterone to renin ratio (ARR). [15, 23] The potential influence of ongoing antihypertensive drugs on ARR results could be significant. Since, in most cases, as all the antihypertensives cannot be stopped safely, testing may be performed while on treatment. However, the mineralocorticoid receptor antagonists (MRA) must be discontinued for at least 4 weeks. [23] Although different values were suggested, the most widely accepted cut-off to define a positive ARR is 30 when aldosterone is measured in ng/dL and PRA in ng/mL/hr [15].

However, along with the evolving perception of PA as a continuum and data supporting variable ARR performance, some authorities recommend the appraisal of aldosterone and renin values independently (or renin and 24-h urinary aldosterone), not just ARR [12, 24].

Finally, if PAC is between 5 and 15 ng/dl, the patient should undergo confirmatory tests. For this purpose, four dynamic tests in use are the oral sodium loading test, the saline infusion test, the fludrocortisone suppression test, or the captopril challenge test, based on local availability [16, 23], and a non-suppressed aldosterone level suggests autonomous renin-independent overproduction.

Once the biochemical diagnosis of PA is made, adrenal imaging should be performed. Most widely, a CT scan is used, followed by MRI. As these visualization techniques fail to distinguish between unilateral and bilateral PA, it is recommended to proceed with adrenal vein sampling (AVS) for disease localization to plan definitive treatment, provided the patient agrees to potentially curative surgery. According to an Endocrine Society Clinical Practice Guideline from 2016, in young patients (below 35 years) with spontaneous hypokalemia, severe PAC, and clear unilateral cortical adenoma on imaging, AVS could be skipped before unilateral adrenalectomy [23]. Recently, there has been growing evidence that AVS still should be performed even in such cases [25, 26].

Unilateral forms of PA can be treated surgically, either via laparoscopic or open approach, while bilateral disease is treated by pharmacotherapy using MRAs, such as spironolactone or eplerenone. Surgical treatment can completely cure hypertension in 30–60% of cases and a marked improvement of BP in the remaining patients, leading to a significant reduction in CV risk [27].

### Pheochromocytoma & Paragangliomas

Phaeochromocytomas and paragangliomas (PPGL) are rare neuroendocrine tumors with an annual incidence of ≈5 patients per million per year [28]. Despite the low prevalence, raising awareness about these tumors is highly important considering the significant clinical impact due to harmful, potentially life-threatening effects, mostly on the CV system, as timely diagnosis and treatment are crucial to obtain the best outcomes. Genetic mutations are detected in ≈70% of cases [29], and PPGL could be the first manifestation of different inherited syndromes such as von Hippel Lindau (VHL) syndrome, multiple endocrine neoplasia type 2 (MEN2), and neurofibromatosis type 1 (NF1).

Clinicopathological features of PPGL are related primarily to the secretion of adrenaline, noradrenaline, and dopamine in various amounts and proportions. As presented in our case, PPGL can also occasionally co-secrete ACTH, cortisol, calcitonin, vasoactive intestinal peptide, testosterone, renin, aldosterone, and interleukin-6 [30]. Also, a multicenter cross-sectional study conducted by Constantinescu et al., provided insight into a new concept of interactions between the adrenal cortex and medulla [31]. The patients included in their study had higher levels of cortisol, 11-deoxycortisol, 11-deoxycorticosterone, and corticosterone than those with primary hypertension, which certainly contributes to the pathogenesis and severity of hypertension in PPGL.

Pheochromocytomas are diagnosed in 0.2–0.6% of patients with hypertension [32], but up to 95% of patients with pheochromocytomas have high BP, while others are normotensive [32, 33]. Hypertension is considered one of the characteristic features, either episodic or constant. It could be the first manifestation of PPGL, even in the form of a catecholamine-induced hypertensive crisis [34]. High BP is mainly due to vasopressor effects of increased levels of circulating adrenaline and noradrenaline secreted by these tumors [31].

Resistant hypertension and hypertension with typical episodic symptoms of catecholamine excess (headache, palpitations, diaphoresis) are indications for biochemical screening for PPGL. With the widespread use of CT scans and MRI for cross-sectional imaging as a diagnostic evaluation, clinicians often encounter a PPGL when assessing the hormonal activity of adrenal incidentalomas (AI). As per the recently published guidelines, an AI tumor radiodensity of > 10 HU should prompt diagnostic evaluation for a pheochromocytoma [35].

A recently published position statement and consensus of the Working Group on Endocrine Hypertension of the European Society of Hypertension recommended that the first-line analysis in screening for PPGL should be measurements of plasma or urinary-free normetanephrine and metanephrine with the addition of 3-methoxytyramine in plasma if a dopamine-secreting tumor is suspected [28]. Imaging evaluation with CT scan or MRI is usually sufficient, but in cases with bilateral adrenal masses, additional functional imaging should be performed (positron emission tomography [PET], or ^123^Iodine meta-iodobenzylguanidine [^123^I-MIBG] scintigraphy) [16].

The therapeutic approach for PPGL consists of preoperative administration of α-adrenergic receptor blockers (phenoxybenzamine and doxazosin) with subsequent β-blocker administration (with or without other antihypertensive agents) for prompt BP control [36] followed by surgical removal of the tumor (laparoscopic or open approach). When the disease cannot be operated, or with the presence of metastatic disease, the options include chemotherapy, radionuclide ablation procedures, or the use of tyrosine kinase inhibitors [2]. Ideally, all cases should be followed up lifelong with biochemical (and imaging when needed) monitoring to screen for recurrence.

### Cushing’s Syndrome

Cushing syndrome (CS), resulting from glucocorticoid excess in the body, is a common sequela of the use of exogenous corticosteroids for various reasons. At times, autonomous overproduction of glucocorticoids can occur in some individuals, resulting in endogenous CS. The pathological hallmark of this type of CS is cortisol excess, either due to adrenocorticotropic hormone (ACTH) overproduction (ACTH-dependent) as in the case of an ACTH-producing pituitary tumor (Cushing disease; CD) or ACTH–independent secretion from an adrenal adenoma. CS, as such, is a rare disease, with an annual incidence of 2 to 8 cases per million population [37]. Rarer forms of CS due to various genetic disorders are also described (beyond the scope of this review and may be referred to elsewhere) [37].

Clinical suspicion for endogenous CS should prompt laboratory evaluation by initial screening tests, including an ODST, 24-h urinary free cortisol (UFC) assay, and/or late-night salivary cortisol (LNSC), depending on local availability [37, 38]. A loss of circadian rhythm with elevated level of cortisol at midnight, high UFC, or failure to suppress cortisol level at 8 a.m. after peroral administration of 1 mg dexamethasone at 11 p.m. the night before requires further biochemical testing and, if CS is confirmed, imaging procedures (CT, MRI, positron emission tomography, bilateral inferior petrosal venous sampling) for tumor localization.

The overall standardized mortality rate (SMR) in Cushing disease (CD) is double than that in the general population. At the same time, coexistent hypertension and diabetes mellitus (DM) are associated with significantly worse survival rates [39]. The leading cause of death is CVD [40, 41], which is of particular concern knowing that patients with cortisol excess have a four- to five-fold higher risk of developing CVD in comparison to the general population [2].

Hypertension is present in approximately 80% of patients with CS [42], independent of their age and sex, and it is multifactorial. It encompasses cortisol binding to the mineralocorticoid receptors with consequent sodium retention and hypervolemia, increased production of endothelin-1 and other vasoconstrictors coupled with inhibition of vasodilator release, but also a modulation of the renin–angiotensin–aldosterone system (RAAS) activity [42–45].

Since there is a correlation between the duration of uncontrolled hypercortisolemia and the development of hypertension [46], hypertension should be treated as soon as it is diagnosed. Considering the pathophysiological aspects of hypertension in CD, Isidori et al*.* proposed a tailored treatment algorithm that uses angiotensin-converting enzyme inhibitors (ACEi) or angiotensin receptor blockers (ARB) as the first-line treatment [47]. If the BP control is not achieved, hypokalemia is the next determining factor and if present, spironolactone or eplerenone should be added, and if absent, calcium antagonists are recommended as add-on therapy. In the third line, alpha-blockers, nitric-oxide donors, and cautious use of diuretics or beta-blockers should be considered.

The surgical removal of the cortisol-producing tumor is the primary therapeutic approach in CS patients, but even with remission of hypercortisolemia, hypertension, and other metabolic disturbances do not necessarily normalize and often persist even during the follow-up period [41]. Upon remission of CS, amelioration in cardiac morphology (like hypertrophy) is frequently detected, but structural changes may not be fully resolved [48]. Persistent hypertension during follow-up has been reported in 25–54% of patients in remission [46] and could be an indirect sign of irreversible cardiovascular changes. Pharmacological therapy in CS also has a positive impact on BP values. Pasireotide reduces BP [49], and a similar effect is gained with other drugs that control hypercortisolism [47].

### Acromegaly and Growth Hormone Deficiency

As per our current knowledge, both growth hormone (GH) excess and deficiency could be associated with hypertension by different pathobiological mechanisms.

Acromegaly is a rare disease with a prevalence between 2.8 and 13.7 cases per 1,00,000 people [50]. The disease is characterized by GH excess, most commonly originating from a pituitary adenoma, and elevated levels of IGF-1, its primary mediator. Typical clinical features include altered facial appearance (large lips and nose, mandibular overgrowth, prognathism, etc.) and acral enlargement. Acromegaly is strongly associated with CV, metabolic, respiratory, musculoskeletal, neurological, and neoplastic comorbidities, not necessarily completely reversible even after full disease control is achieved [51, 52].

For many years, CVD was the main cause of mortality in patients with acromegaly. Based on raised awareness among clinicians in recent years, a shift to cancer as the leading cause is notable [53]. Hypertension is the most frequent CV manifestation in acromegaly, with an estimated prevalence of about 30% [54]. However, in some cohorts, the prevalence reached 56% at study entry and 64% during the follow-up [55]. Pathogenesis of hypertension is multifactorial, including increased serum GH levels leading to insulin resistance due to the decreased insulin effects on liver and extrahepatic tissues, endothelial dysfunction, increased renal sodium reabsorption, and consequent expansion of extracellular volume, and sleep apnea syndrome [56].

A current therapeutic approach to acromegaly should aim not only for the biochemical normalization of IGF1 levels but also for optimal control of comorbidities and complications [57]. Since hypertension in acromegaly is usually mild, the Acromegaly Consensus Group recommends that treatment guidelines for hypertension for the general population should also be implemented in patients with GH excess [54].

As previously mentioned, GH deficiency can also represent a risk factor for the development of hypertension. Since GH highly influences the proportion of lean body mass and adipose tissue, patients with low GH and IGF1 levels have a risk CV risk profile. While a peak of GH secretion occurs in puberty, the decline afterward is remarkable: every 7 to 10 years, the secretion drops by 50% so that at the eighth decade, the serum GH levels are comparable to those among GH-deficient young adults [58]. While in children and adolescents with proven GH deficiency (GHD), the benefits of GH as replacement therapy are clear, including improvement of hypertension, in older individuals, the potential adverse effects (primarily the risk of development of malignancy) limit its use and therefore, the role remains controversial.

### Thyroid Disease and Hypertension

Thyroid disorders are widespread, with an estimated prevalence of more than 10% in the adult population [59], but they are held responsible for hypertension in just about 1% of patients [60]. Considering such a small proportion, measurement of thyroid hormones (TH) is not routinely advised in otherwise asymptomatic patients. Thyroid dysfunction greatly influences CV risk through the direct effects of TH on myocardial and vascular cells and indirectly by impairment of lipid profile and raising systolic and/or diastolic BP. [61, 62]

#### Hypothyroidism

A connection between hypothyroidism (high thyroid-stimulating hormone (TSH) with low free thyroxine level (fT4) and elevation of diastolic BP (DBP) has been recognized for a long time [63, 64]. An 11-year follow-up study in 14353 individuals, however, showed that not only DBP is higher with every 1 mU/L increase in TSH levels (mean increase of 1–2 mmHg), but also systolic BP (SBP) rise of approximately 2 mm Hg [65].

High levels of TSH are associated with endothelial dysfunction, increased arterial stiffness, renovascular resistance, hypercoagulable state, and an atherogenic profile of lipid parameters [66, 67]. According to that, a recent meta-analysis conducted by Ding et al. [68] revealed that not only overt but also subclinical hypothyroidism (SCH), defined as normal serum fT4 with increased TSH level, is associated with hypertension (pooled OR of 1.76) together with other three out of four components of metabolic syndrome (MetS). Another meta-analysis of 19 prospective follow-up studies showed that therapy with levothyroxine (LT4) applied to patients with SCH reduces SBP (mean difference − 4.80 mmHg) and DBP (mean difference: − 2.74 mmHg). [66] LT4 therapy had a higher influence on lowering SBP in patients with higher SBP baseline values.

#### Hyperthyroidism

Hyperthyroidism, defined as a suppressed TSH level with an excess of TH, is characterized by elevated SBP and widened pulse pressure. In cases of thyrotoxicosis, the estimated frequency of hypertension is high, 20–68% [60]. TH strongly influence heart rate, based on their effects on β-adrenergic receptors on myocytes, leading to increased cardiac output, but also decreases peripheral vascular resistance by about 70% via vascular smooth muscle [67]. Other mechanisms involved include arterial stiffness and higher pulse wave velocity, increased levels of renin, angiotensin, and aldosterone by activation of the juxtaglomerular apparatus in the kidney, high levels of endothelin-1, stimulation of erythropoietin secretion [67]. A study by Iglesias et al. demonstrated that successful treatment of hyperthyroidism decreases SBP values approximately by 5 mm Hg [69].

### Primary Hyperparathyroidism

Primary hyperparathyroidism (PHPT) is nowadays not a unique disease but a spectrum of various forms: from classic entity characterized by excessive secretion of parathyroid hormone (PTH) coupled with hypercalcemia to the normocalcemic hyperparathyroidism (NPHPT) recently becoming increasingly recognized.

The reported prevalence of hypertension in PHPT is high, from 40 to 65%. [70] Although the precise mechanisms are not completely understood, both calcium and PTH have effects on the cardiac muscle and vascular smooth muscle cells [71] but also affect BP via the RAAS [72] and increased endothelin 1 [73].

A recent study conducted by Karwacka et al*.* showed a decrease in BP in approximately 78% of patients with concomitant hypertension after parathyroidectomy [74]. SBP and DBP were reduced by 12 and 6 mm Hg, respectively, at six months follow-up. Similar results were obtained by Karakose et al*.* [75]*,* pointing out that we should consider additional parameters when choosing the appropriate treatment modality for patients diagnosed with PHPT, particularly in the early phase of the disease. Furthermore, parathyroidectomy is independently associated with a reduced need for antihypertensive medications in the future and lesser chances for the introduction of drugs at all periods in patients without previously elevated BP [76].

Despite the new findings and because of conflicting data based on the association of PHPT with other CV risk factors, according to current guidelines, hypertension is not a point of reference when deciding on surgery [77]. Moreover, a recent Cochrane review of RCTs on parathyroidectomy in patients with asymptomatic PHPT did not show significant CVD benefits. However, the studies included in the review were small and mostly with short follow-up periods [78].

### Renal Artery Stenosis and Other Rare Forms of Renin-Driven Hypertension

Renal artery stenosis (RAS) arising from the atherosclerotic process is the most common form of renovascular hypertension. Infrequently, RAS also occurs from fibromuscular dysplasia of the renal artery. Rarely, renal artery dissection, aneurysm, systemic vasculitis, arteriovenous fistula, or embolic disease can also cause renovascular hypertension [79]. A criterion of hemodynamically significant RAS, according to guidelines, is a decrease of a minimum of 60% in the diameter of the renal artery [80]. Since atherosclerosis is the most common etiology, RAS is frequently encountered in the older population.

Renal hypoperfusion causes alterations of the kinin-kallikrein, endothelin, and sympathetic nervous systems [81]. Still, the most prominent sequel is the activation of RAAS, a cornerstone of circulatory homeostasis, with the release of renin from juxtaglomerular cells as the first step. Renin consequently leads to the formation of angiotensin I and angiotensin II, which are associated with a wide range of biological effects in the heart, vascular system, kidney, brain, and immune system [82]. Furthermore, angiotensin II triggers the release of aldosterone from the adrenal cortex, which boosts tubular sodium reabsorption, resulting in plasma volume expansion.

Treatment options mostly rely on conservative treatment with antihypertensive, hypolipidemic, and antiplatelet agents, while renal revascularization procedures are used in specific, individually assessed clinical situations [80]. The recommendation is based on the results from worldwide studies that did not prove a clear benefit of percutaneous angioplasty (with or without stent) or surgery. A previous meta-analysis, including 8 studies involving 2223 participants, showed no association between renal artery revascularization and a change in SBP or reduced adverse CV or renal outcomes compared to a conservative treatment strategy [83]. The only benefit was a slight reduction in the number of medications required to control hypertension during the follow-up period.

Much rarer causes of renin-driven hypertension include Page kidney resulting from an external kidney compression, which activates RAAS, and reninoma, a benign tumor of juxtaglomerular cells with only ≈100 cases published to date [16].

### Sleep Apnoea and Hypertension

Obstructive sleep apnea (OSA) is characterized by recurrent periods of upper airway collapse, which cause obstruction and, consequently, intermittent reduction in arterial oxygen saturation. The diagnosis is made by polysomnography, which is performed when OSA is suspected based on clinical symptoms such as daytime sleepiness and fatigue, problems with concentration or headaches, snoring, or periods of apnea reported during sleep.

Traditional risk factors for the development of OSA encompass male gender, obesity, smoking, and craniofacial or oropharyngeal anatomic abnormalities [84]. Besides them, studies have established that increasing age, overweight, alcoholism, higher Epworth sleepiness scale score, mean apnea duration, oxygen desaturation index, and nocturnal oxygen desaturation are additional factors [85].

Based on its relationship with CVD, such as hypertension, stroke, HF, coronary artery disease, and atrial fibrillation [86] and the obesity pandemic, OSA has gained much more clinical significance in the last decade. According to current knowledge, potential links include sympathetic overactivity, gut dysbiosis, and proinflammation [87], but also activation of RAAS and hyperaldosteronism [88, 89].

In clinical practice, OSA is often associated with resistant hypertension, which is hard to control with 3 or more antihypertensive drugs. Based on studies, OSA can be found in up to 70% of patients with resistant hypertension compared to 38% of those with essential hypertension [90]. Therefore, individuals with resistant hypertension should be considered to have screening evaluation for OSA when clinically suspected [86, 87, 91]. Prompt treatment of OSA has been associated with significant improvements in hypertension [91].

### Rare Forms of Endocrine Hypertension

Several uncommon clinical and genetic conditions are associated with hormone-mediated increases in BP. A detailed discussion of these rare forms of endocrine hypertension is beyond the scope of this review. Therefore, a summary of these conditions, their genetic defects, important clinical features, and management are discussed in the Table 2 below:

**Table 2:** Rare causes of endocrine hypertension, their genetic defects, type of inheritance, endocrine/metabolic abnormalities, common clinical features, and management options [92–94]. *ACTH* = *Adrenocorticotrophin; AD* = *Autosomal Dominant; AME* = *Apparent Mineralocorticoid Excess; AR = Autosomal Recessive; CAH* = *Congenital Adrenal Hyperplasia; CCB* = *Calcium Channel Blocker;* *FHHt* = *Familial Hyperkalemic Hypertension; HTN* = *Hypertension; IUGR* = *intrauterine growth retardation; JGCT* = *Juxta Glomerular Cell Tumor; MRA* = *mineralocorticoid receptor antagonist; POR* = *Cytochrome P450 oxidoreductase*

Disease entity	Defective gene	Inheritance pattern	Hormone/metabolic abnormalities	Common clinical picture	Treatment options
AME	*HSD11B2*	AR	Low renin, low aldosterone, hypokalemia and metabolic alkalosis	IUGR, failure to thrive, severe HTN; Nonclassical-AME: miId HTN with onset in adulthood	MRA, thiazide, low salt diet
CAH due to 11ß-hydroxylase deficiency	*CYP11B1*	AR	Low renin, low aldosterone, hypokalemia and metabolic alkalosis	Early onset of hypertension, virilisation (46 XX) and raised 17-hydroxyprogesterone levels	Glucocorticoids to suppress the ACTH from pituitary & anti-hypertensives
CAH due to 17-α hydroxylase deficiency	*CYP17A1*	AR	Low renin, low aldosterone, hypokalemia and metabolic alkalosis	Early onset of hypertension, ambigucus genitalia(46XY), low 17- hydroxyprogesterone levels	Glucocorticoids, sex hormone replacement and MRA when needed for HTN control
CAH from POR deficiency	*POR*	AR	Low renin, low aldosterone, hypokalemia and metabolic alkalosis	Early onset of hypertension, ambiguous genitalia (46XY), low 17- hydroxyprogesterone levels	HTN control with MRA, Amiloride, or CCB
Liddle syndrome	*SCNN1B, SCNN1G, or SCNN1A*	AD	Low renin, low aldosterone, hypokalemia and metabolic alkalosis	Severe HTN of early onset in childhood that does not respond to MRA treatment	Usually respond to low salt diet and Amiloride
Geller syndrome	*NR3C2*	AD	Low renin, low aldosterone, hypokalemia and metabolic alkalosis	Early-onset of severe HTN which gets worse with MRA treatment and during pregnancy	Managed by low salt diet and amiloride
Chrousos syndrome	*NR3C1*	AD	Low renin, low aldosterone, hypokalemia and metabolic alkalosis	High serum cortisol and ACTH levels without features of Cushing syndrome	ACTH suppression with high doses of glucocorticoids
JGCT	*p53,* *Deletions of Rb gene*	Polygenic	High renin and aldosterone levels with hypokalaemia and metabolic alkalosis	Refractory HTN in adolescence or young adults (usually in females)	Surgical removal of the tumor. Aliskiren is useful for HTN
Gordon syndrome or FHHt	*WNK4, WNK1, KLHL3, CUL3*	AD	Low renin, normal aldosterone with hyperkalaemia and metabolic acidosis	Hyperkalemia and type-4 renal tubular acidosis in patients with normal renal function	Low salt diet and thiazide diuretics

## Conclusion

Despite all the efforts from the global scientific community to curtail the high prevalence, hypertension remains a leading cause of morbidity and mortality from CVD. It is prudent to consider an underlying SH, especially in patients presenting with early-onset, resistant, or severe hypertension, particularly in those with suggestive clinical clues like hypokalemia. Furthermore, incidentally discovered adrenal lesions require minimum testing with ODST and ARR if they have hypertension, with the addition of plasma/urinary metanephrine analysis depending on the radiodensity of the lesion. In addition, a family history of early-onset hypertension or stroke should raise attention and demand a basic examination.

**Fig. 4:**
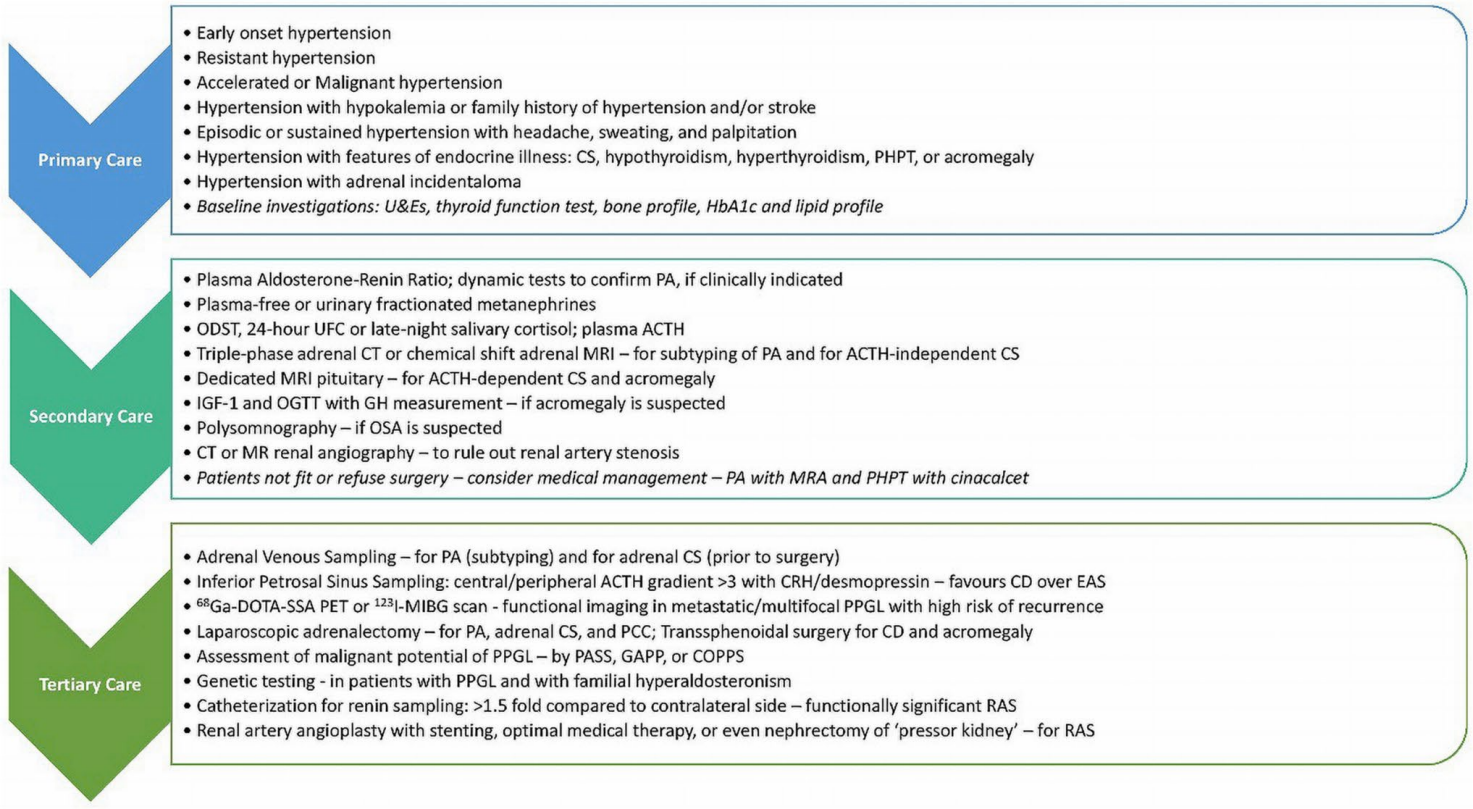
An approach to evaluation and management of endocrine hypertension (adapted from Fernandez et al.) [2]

Endocrine hypertension represents a cluster of disease entities and requires vigilance and proper use of screening and confirmatory testing. If diagnosed promptly and intervened early based on appropriate disease-specific therapy, some forms of endocrine hypertension could be completely cured. If complete relief from hypertension cannot be achieved, the institution of a proper treatment strategy allows better control of BP and prevention of long-term complications like target organ damage and premature mortality. A pragmatic and systematic approach to evaluating and managing endocrine hypertension is summarised in Fig. 4.

## Key References

- Fernandez CJ, Nagendra L, Alkhalifah M, Pappachan JM. Endocrine hypertension: The urgent need for greater global awareness. touchREV Endocrinol. 2023;19(2):31–41. https://doi.org/10.17925/EE.2023.19.2.11.
  - This review emphasizes the need for raised consciousness regarding endocrine causes of hypertension and its clinical significance. The paper also summarises the practical approach diagnostic evaluation and management of endocrine hypertension.
- Turcu AF, Nhan W, Grigoryan S, Zhang L, Urban C, Liu H, Holevinski L, Zhao L. Primary aldosteronism screening rates differ with sex, race, and comorbidities. J Am Heart Assoc. 2022;11(14):e025952. https://doi.org/10.1161/JAHA.122.025952.
  - Findings from this study suggest that screening for primary aldosteronism is being considered with delay and just in a small subgroup of high-risk patients, mostly in ones with hypokalemia and multiple comorbidities.
- Stowasser M, Jansen P, Wolley M. Systematic approach to the diagnosis and management of endocrine hypertension. In: Pappachan JM, Fernandez CJ, editors. Endocrine Hypertension: From Basic Science to Clinical Practice. Eastbourne, UK: Elsevier; 2022. p. 331–68.
  - A textbook chapter that gives the most comprehensive and concise approach to patients with endocrine hypertension. Professor Stowasser, the first author of this article has the largest data of primary aldosteronism in the world and has been author to most international guidelines on primary aldosteronism.
- Fassnacht M, Tsagarakis S, Terzolo M, Tabarin A, Sahdev A, Newell-Price J, et al. European society of endocrinology clinical practice guidelines on the management of adrenal incidentalomas, in collaboration with the European Network for the Study of Adrenal Tumors. Eur J Endocrinol. 2023;189(1):G1-G42. https://doi.org/10.1093/ejendo/lvad066.
  - A recently published guideline provided the optimal diagnostic work-up for frequently encountered adrenal incidentalomas, particularly in individuals with hypertension, and pointed out the clinical utility of initial determination of tumor radiodensity.
- Brown J, Yazdi F, Jodari-Karimi M, Owen JG, Reisin E. Obstructive sleep apnea and hypertension: Updates to a critical relationship. Curr Hypertens Rep. 2022;24(6):173–184. https://doi.org/10.1007/s11906022–01181-w.
  - A clinical update article linking the relationship between sleep apnea and hypertension. This important interlink explained well in this paper empowers clinicians to have better understanding about this problem grossly undermanaged in clinical practice due to poor awareness.

## Data Availability

No datasets were generated or analysed during the current study.
